# Illinois State Medical Society

**Published:** 1873-06-15

**Authors:** 


### Society Reports.

### ILLINOIS STATE MEDICAL SOCIETY.

The twenty-third annual meeting of the Illinois State Medical Society convened in Bloomington, May 20, 1873.

The delegates and members assembled in Durley Hall at 10½ A.M. The President, Dr. D. W. Young, of Aurora, occupying the chair, called the meeting to order, and introduced Rev. Dr. Wilson, who offered prayer.

Dr. T. F. Worrell, Chairman of the Committee of Arrangements, was then introduced, and delivered a brief address of welcome in behalf of the citizens of Bloomington. This was responded to, in behalf of the Association, by Prof. E. Andrews, of Chicago.

Brief addresses of welcome were also offered by Ira J. Bloomfield, on behalf of the municipal authorities; on behalf of the Board of Education, by J. A. Jackman, Esq., which were responded to by members of the Society, after which the meeting adjourned until 2 o'clock, P.M.

On re-assembling at 2 o'clock the Society proceeded to business. The Committee on Credentials made their report, and the roll of officers of committees was called by the Secretary.

The first paper read was the report on Surgery, by J. L. White, M.D., of Bloomington. This was an able and interesting report, and elicited considerable discussion.

Dr. E. Andrews thought the remarks made on the subject of fractures of the bones of the hips were of considerable importance. The books were quite deficient in distinguishing these injuries, or the different grades or complications of them from each other, so that when a practitioner meets one he is perplexed as to what prognosis he is to give. These injuries are not of frequent occurrence; still, every once in a while some of us meet them. His own observation had been, without exception, as far as he could recollect, that all cases of fracture of the pelvic bones, accompained by serious wounds of the viscera, followed the usual course mentioned in the books—that is, have died. With regard to the cases where the viscera are not wounded, the books were not clear. He had seen a number of these cases, even severe ones, in which the viscera had escaped, and every one of them was living to-day unless they had died recently.

One case in particular, a young man, who, in attempting to jump upon a train of cars in motion, was rolled over and over in front of the wheels. The first thing noticeable, in examining the case, was the dislocation of the head of the femur, backward on the right side. This was reduced by manipulation. On examining for further injuries it was found that on pressing upon the crests of the ileum they could be pushed toward each other an inch and a half, showing that there was a serious fracture. No unfavorable symptoms occurred, however, and he made a good recovery. In several other cases the mobility of the parts was quite as distinct, and still the patients recovered. Judging from his own experience, therefore, he should say that we ought to make this distinction: That fractures of the pelvis, accompanied by wounds of the viscera, are nearly invariably fatal; while those not accompanied by wounds of the viscera, almost as invariably recover.

Dr. D. Prince, of Jacksonville, said that he had noted some points in the report in relation to which he would like to say a few words. First, he said, in regard to union by first intention, he always, after amputations, put the flaps in position, and allowed them to remain. Whether there was a deposition of new material between their surfaces or not, was a theoretic question. But that the flaps would stick together with considerable force, was abundantly proved by attempting to separate them. He thought, therefore, that it was wrong to wait six or twelve hours, as recommended in the report, before bringing the flaps together. A better way was to put the flaps in position, and then, if they fully united, nothing would be lost. If there should be an accumulation so as to necessitate opening them again, nothing then would be lost.

Again, in regard to the subject of shock, Dr. Prince remarked that the nervous functions were greatly depressed by shock. It produces a vital impression, different from that which is the result of a mechanical injury to the nervous system. Shock is vital. A person takes a glass of ice-water, and falls down almost pulseless—skin cold, and bathed in perspiration—it is a shock. But a severe blow is received, as when a person is crushed beneath a car-wheel, and the whole nervous system is involved in the shock. Prostration should apply to a condition coming on slowly. In the case of shock, what should be done? It is well known that any stimulant diminishes the feeling of nervous exhaustion for a time. Whatever theory of alcoholic action is admitted—whether it nourishes or whether it takes the place of something removed by nutrition—it is difficult to see how any quantity of alcohol introduced into the system will be injurious. The report admits that all stimulants are not to be excluded, for opium is admitted, and I presume in stimulant not narcotic doses. The exclusion of light from the patient, the reserving him from the fatigues attendant upon being moved, excluding him from conversation or any act of attention, are all in accordance with correct reasoning; but I cannot see why the patient is not to take stimulants directly, without exciting irritability. This question of the use of stimulants in shock was discussed, pro and con, at considerable length.

Dr. Andrews remarked, in regard to the subject, that the first step toward knowledge is for the profession to understand what it does not know. I believe that every thoughtful surgeon has concluded, before this time, that among the things in which our knowledge is wretchedly deficient is the relations of therapeutics to shock. Almost everything that we can do has two edges to it. The discussion of this subject in associations and in books all proceeds theoretically. There exists nowhere any adequate collection of statistics that will enable us to ascertain scientifically what the effect of treatment upon shock is. I have been in the habit of considering an ordinary shock to mean an impression upon the ganglionic nervous system, which controls the circulation and the organic functions of life.

A cerebral shock proper is that which we have in a concussion of the brain. Now, what is the effect of a stimulant in such a case? is what we want to know. I am always in the habit of giving stimulants; but I have wished many times, and wish now, that I knew better whether the stimulants do the patient any good or not.

We may speak of shocks as divided into three classes. There is a class, as you know, which are not dangerous; there is another class in which it makes no difference what you do; medicine has no effect. It is to a third class, dangerous, but not necessarily fatal, that our treatment is mainly confined. Dr. Prince has presented the argument on one side, and the report of the Committee has brought out the other. The argument cuts two ways. Almost all alcoholic stimulants, and nearly all others, cause an increase of the force of the pulse temporarily, and the functions of the nervous system which we particularly wish to increase. The nervous system is exalted; but afterwards there is a falling off in diminution; the patient is first raised above, and then depressed below the natural condition. Is that what we want? One party says we must avoid stimulants, in order to avoid this depression. The other says, we must use them to get the elevation. What shall we say about the application of this doctrine? I use stimulants; but I wish it was scientifically made more clear that it is the right thing to do. At present we are driven to discuss the question from a purely theoretical point of view. I think a practical solution of the question might be made if all practitioners would adopt this plan: Whenever a case of injury occurs, let all men who think stimulants are beneficial observe carefully the effects upon the patient as derived from his reports of himself. While not exactly a scientific demonstration, it would lead us in the right direction.

Dr. Andrews inquired if any one had used oxygen inhalations in shock. He understood that Dr. McGraw, of Detroit, had been more successful in using it than with other stimulants.

Dr. Ingalls, of Chicago, thought that there was one radical error in Dr. Andrew's views in relation to the use of stimulants. He seemed to adopt the idea that the vital powers, being raised by stimulants to a certain degree were then depressed to a corresponding degree. From his own observation he did not think it was true—that was when properly administered. When an alcoholic stimulant had produced stimulation, and then is followed by depression, the alcoholic stimulation is indiscreet. After some further remarks from Dr. White, of Bloomington, opposing the use of stimulants, the report was finally referred to the Committee on Publication.

Dr. Hamilton next presented a report on Fractures, supplementary to the report on Surgery. It was an extended paper, mainly made up of statistics.

Dr. E. L. Holmes read the report of the Committee on Opthalmology. The Secretary also read an interesting paper on the uses of Strychnia, prepared by Dr. F. C. Holtz, of Chicago.

The Society then adjourned until 7½ P.M., when they re-assembled to listen to the address of Dr. Geo. T. Allen, on the "Civilization of the Age."

Following this address, Dr. McFarland read a paper devoted mainly to the subject of the Jurisprudence of Insanity.

The Society re-assembled again at 8½

o'clock, A.M. The first business in order was the report of the Treasurer, Dr. Hollister.

The report of Dr. Hamilton, on Fractures, was again called up and discussed at length by various members, particularly the points in relation to the possibility of preventing shortening in oblique fractures of the femur. It was the pretty unanimous opinion of the members that some degree of shortening would almost invariably and unavoidably result, although the statistics given in the report of Dr. Hamilton seemed to favor a contrary opinion.

The report on Drugs and Medicines, by Dr. Hollister, was next read and discussed. Dr. Prince, of Jacksonville, also submitted a report on Galvano-Therapeutics.

The Committee on Nominations submitted the following report:

For President, Dr. T. F. Worrell, of Bloomington; 1st Vice-President, Dr. E. L. Holmes; 2d Vice-President, Dr. Birney; Treasurer, Dr. J. H. Hollister. Next place of meeting Chicago.

*Committee on Practical Medicine*—Dr. H. Crawford, Monmouth; Dr. John Wright, DeWitt; Dr. Von Horn, Jerseyville.

*Surgery*—Drs. W. P. Pierce, P. Raskatin, E. R. Willard.

*Obstetrics*—Drs. S. C. Plumber, C. T. Hassian, Robert Beal.

*Drugs and Medicine*—Drs. W. E. Quine, Chicago; R. W. James, Danville; E. P. Cook, Mendota.

*Ophthalmology*—Drs. E. L. Holmes, J. P. Johnson, F. C. Holtz.

*Otology*—Drs. S. J. Jones, Chas. Hunt, Thos. Galt.

*Special Committee on Idiocy*—Dr. C. F. Wilber.

*Galvano-Therapeutics*—Dr. D. Prince

*Pathology of Nervous System*—Dr. C. W. Earle.

*Dermatology*—Dr. Chas. G. Smith.

*Phthisis Pulmonalis*—Dr J. H. Hollister.

*Strictures of Urethra*—Prof. Andrews.

*Diseases of Respiratory Organs*—Daniel Lichty.

*Committee of Arrangements*—Drs. N. S. Davis, W. E. Quine, Chas. G. Smith, E. Powell.

*Assistant Secretary*—Dr. W. E. Quine.

The report of the Nominating Committee was unanimously adopted.

A report on "The Assistance necessary and justifiable in Difficult and Protracted Labor," by Dr S. D. Hawley, was read by the Secretary. The report elicited extended and various criticism and discussion, which space does not permit us to report. The Society again adjourned until evening. The afternoon being spent, at the invitation of the Committee of Arrangements, in visiting the State Normal School, the Soldiers Orphans' Home, and the C. A. & St. L. R. R. machine shops. At each of these places the Society was welcomed by the authorities in charge.

The Association re-assembled in the evening session at 8 o'clock, P.M.

Dr. Worrell read a report upon Hygiene, and in connection with it a supplementary report, prepared by Dr. N. S. Davis, on the Hygienic Management of Children.

A report on the Physiology of the Nervous System, by Dr. Earle, the report on Otology, by Dr. S. J. Jones, and a report on Obstetrics, from Dr. T. D. Fitch, completed the list of reports presented.

On motion of Dr. Hollister, the Association proceeded to the election of delegates to the American Medical Association, the following named being chosen:

Drs. A. H. Luce, J. O. Hamilton, E. Goodbrake, E. P. Cook, Dr. Pierce, Dr. Prince, C. T. Homer, J. H. Hollister, W. W. McMann, S. H. Birney, C. W. Earle, J. B. Walker, Dr. Haller, W. E. Quine, Dr. Willard, Dr. Whitmeyer, E. R. Travers, J. P. Mathews, E. W. Hewett, D. Young, T. D. Fitch, O. W. Moon, John Lyttle, A. C. Rankin, O. Everett, F. H. Davis, J. G. Curtis, S. E. Plummer, F. M. Wilder, J. L. White, A. S. McArthur, N. B. Cole, D. McVey, C. Armstrong, and Dr. McFarland.

Dr. Fitch moved that the Association proceed to the election of delegates to State Medical Societies. The motion was carried, and the following persons were elected:

Drs. G. W. Jones and Birney, to Indiana Medical Society; Drs. Gray and Ellsbury, to Ohio; Drs. M. Gunn and E. L. Holmes, to Michigan; Drs. McArthur and Irwin, to Wisconsin; Drs. T. D. Fitch and J. Galt, to Iowa; Drs. C. R. Cook and J. O. Hamilton, to Missouri; Drs. T. F. Worrell and E. R. Willard, to Minnesota.

A motion from Dr. Hollister that the annual assessment be fixed at $3 for the year, was carried.

After passing resolutions of thanks to the Committee of Arrangements, the municipal authorities, and others who had assisted in entertaining the Association, the meeting was adjourned, *sine die.*

---

HOPS AS AN EXTERNAL ANODYNE.—Enclose the hops in a bag, and subject it to the steam of boiling water, and apply as warm as can be borne. Dampening the hops *slightly* with strong vinegar before steaming increases their anodyne virtues.

